# Effect of Intrauterine Infusion of Equine Fresh Platelets-Rich Plasma (PRP) or Lyophilized PRP (L-GF^equina^) on Ovarian Activity and Pregnancy Rate in Repeat Breeder Purebred Arabian Mares

**DOI:** 10.3390/ani11041123

**Published:** 2021-04-14

**Authors:** Ahmed Dawod, Jordi Miro, Hamed T. Elbaz, Hossam Fahmy, Ahmed S. Abdoon

**Affiliations:** 1 Husbandry and Animal Wealth Development Department, Faculty of Veterinary Medicine, University of Sadat City, Menofia 32897, Egypt; adawod1980@gmail.com; 2 Department of Animal Medicine and Surgery, Veterinary Faculty, Autonomous University of Barcelona, E-08193 Barcelona, Spain; 3 Theriogenology Department, Faculty of Veterinary Medicine, University of Sadat City, Menofia 32897, Egypt; hamed.elbaz@vet.usc.edu.eg; 4 Faculty of Medicine, Ain Shams University, Cairo 11341, Egypt; hossamfahmy@gmail.com; 5 Department of Animal Reproduction and Artificial Insemination, National Research Center, Giza 12622, Egypt

**Keywords:** L-GF^equina^, fertility, Arabian mares, estrus cycle length, pregnancy rate

## Abstract

**Simple Summary:**

Persistent endometritis is one of the major causes of infertility in mares and results in endometrium degeneration, namely, endometrosis. Platelets-rich plasma (PRP) is an emerging therapeutic application in tissue regeneration because of its high concentration of growth factors with a high mitogenic and anti-inflammatory potential, but reduced preservation time. L-GF^equina^ is a freeze-dried, stabilized, platelet-rich plasma product (PP) that can be stored at 2 to 8 °C for several months. It can be easily used after reconstitution with sterile saline or water, eliminating the need for repeated blood product collection and special equipment. The intrauterine infusion of equine platelet-rich plasma or L-GF^equina^ can be used indistinctly in repeat breeder mares, shortening the estrous cycle, with no significant effect on the follicular diameter, as well as increasing pregnancy rate in Arabian purebred mares.

**Abstract:**

This study was designed to examine the effect of the intrauterine infusion of platelet-rich plasma (PRP) or equine lyophilized growth factor (L-GF^equina^) on the follicular growth, endometrial thickness, estrus cycle length, and pregnancy rate in purebred Arabian mares. A total of 73 purebred Arabian mares who experienced repeat breeding for three successive cycles were randomly divided into the following three groups: control group, without treatment; second Group (PRP group), in which mares were intrauterine infused with 20 mL of fresh PRP on the second day after the end of physic estrus phase; and the third group (L-GF^equina^ Group), consisting of mares that were intrauterine infused with 20 mL of reconstituted lyophilized horse platelets growth factors (L-GF^equina^) on the second day after the estrus phase. The results showed no significant difference between control and treated groups in the diameter of the preovulatory follicles during the post treatment cycle. The endometrium thickness increased significantly in the L-GF^equina^ and PRP groups ahead of the non-treated group. Intrauterine L-GF^equina^ or PRP administration shortened the estrus cycle length. A higher pregnancy rate was recorded in the L-GF^equina^ and PRP treated mares. In conclusion, the intrauterine infusion of L-GF^equina^ or PRP increased the endometrial thickness and pregnancy rate and could be used to improve fertility in Arabian purebred mares who experienced from repeat breeding.

## 1. Introduction

Despite their economic importance as racing and show animals, Arabian horses constitute an important role in Arabian heritage. The Arabian is not just a pretty horse, but it has unique anatomical, physical, behavioral, and intelligence characters. The sincere, willing nature of the Arabian horse allows it to be the ideal family horse. Its affectionate personality also makes this horse extremely suitable for children [1].

However, pure Arabian horses are facing a marked reduction in their population size because of certain factors such as exportation and diseases. The subfertility of the Arabian horse breed is a great problem facing the expansion of Arabian horse breeding programs, as well as the high prices for this horse breed. Persistent post breeding induced endometritis (PBIE) is one of the major causes of infertility in mares, affecting and hindering their breeding programs [2]. In fertile mares, the mucosa of the female reproductive system uses a defense mechanism that removes contaminants through a combination of cellular, humoral, and mechanical factors of contraction, as well as lymphatic drainage, which facilitates uterine clearance and the elimination of the physiological inflammatory process [3]. However, in susceptible mares, after natural or artificial insemination, persistent breeding induced endometritis (PBIE) is a consequence of the failure of the uterine mechanism to remove the excess spermatozoa, bacteria, or inflammatory products from the uterus [4].

Traditionally, different approaches have been used to treat PBIE in mares, including the systemic or intrauterine administration of antibiotics [5,6]; ecbolics, such as oxytocin or cloprostenol [6]; and the use of non-steroidal anti-inflammatory drugs [7,8]. In addition, the intrauterine infusion of antiseptics (i.e., Lugol’s solution) has been tested [9]. Unfortunately, a subset of mares fails to respond to traditional therapeutics because of their lack of efficacy [10,11], the increasing incidence of antibiotic-resistant bacteria, or their side-effects on the future fertility of the mare [12]. These side effects have led to the development of alternative therapies for mares experiencing PBIE [11].

Currently, platelet-rich plasma (PRP) is an emerging therapeutic application in tissue regeneration because of its high concentration of growth factors with a mitogenic and anti-inflammatory potential [13,14]. PRP is a concentration of platelets containing multiple growth factors that are very important for regeneration and anti-inflammatory processes. Treatment with platelet-rich plasma (PRP) has been used for various conditions in equine veterinary medicine, including in orthopedic surgery; for the repair of muscles, tendons, and ligaments; and for the reversal of skin ulcers [14]. For the treatment of endometritis in horses, the administration of PRP at the time of breeding decreases the intrauterine inflammatory response in mare’s suffering from chronic endometritis [15]. In addition, PRP administration decreases polymorphonuclear neutrophil (PMN) numbers in the uterine lumen and increases pregnancy rates [16]. However, there is a high variability in responses between different mares and between different PRP preparations methods, making the clinical outcome of intrauterine administration uncertain [17]. Therefore, the possibility of preparing a lyophilized PRP is an attractive process. L-GF^equina^ is a freeze-dried, stabilized, platelet-rich plasma product (PP) that can be stored at 2 to 8 °C for several months. In L-GF^equina^, the breakdown of platelets in vitro prior to lyophilization leads to the release of a supra-physiological concentration of growth factors. Among these are transforming growth factor, platelet derived growth factor, fibroblast growth factor, and thromboplastin. These growth factors stimulate the proliferation of several cell lines, including epithelial cells and fibroblasts. It can be easily used after reconstitution with sterile saline or water and eliminates the need for repeated blood product collection and special equipment. Lyophilized platelets have been investigated for wound repair with encouraging results [18,19]. However, little is known about their biological effects regarding the treatment of PBIE in Arabian mares. Therefore, the aim of this study is to compare the effect of intrauterine infusion of fresh or lyophilized PRP on the estrous cycle, follicular diameter, endometrial wall thickness, and pregnancy rates in purebred Arabian mares experiencing repeat breeding.

## 2. Materials and Methods

### 2.1. Animals

This study was conducted in three private studs farms located in the Giza, Gharbia, and Behera provinces, in Egypt, from October 2019 to April 2020. Seventy-three purebred Arabian mares (Asil and Abian strain) were used in the present work, with ages ranging from 5 to 18 years, parity (1–8), and presenting a good body condition (BCS; 5 to 8). All of the mares had a previous history of infertility and repeat breeding, with normal conformation of the external genitalia and with apparently healthy reproductive organs and no fluid accumulation in the uterine lumen. The mares were maintained under uniform management practices and were fed a breeding ration of 14% crude protein obtained from a commercial ration manufacture company (Cairo Feed Company, Giza, Egypt). The females were housed in individual stables with bedded floors and self-serve waterspouts.

### 2.2. Preparation of PRP

The platelet-rich plasma was prepared according to the method described by Carmona et al., 2007. Briefly, 100 mL blood samples were collected from each animal from the jugular vein in tubes containing 3.2% sodium citrate as an anticoagulant. Then, the blood samples were homogenized and centrifuged at 120× *g* for 10 min. After centrifugation, the upper 50% of the plasma was discarded from the centrifugation tubes and the remaining plasma was aspirated with a sterile syringe transferred to another plastic tube without anticoagulant, and was re-centrifuged again at 240× *g* for 10 min. The supernatant was discarded, and the remaining portion was used as the PRP. Then, the plasma was maintained at 20–25 °C in an isothermal box for 1 h until application. The activation of the platelets was done immediately before intrauterine infusion to release their platelet granules via a calcium chloride solution at a dose rate of 0.068 mEq calcium for each mL of PRP [20]. The average concentration of platelets was 2 × 10^6^ platelets/µL.

### 2.3. Lyopholoized L-GF^equina^

L-GF^Equina^ consists of lyophilized horse platelets growth factors. It is produced by a patented method developed by D. Hossam M. Fahmy. Each individual unit of pheresis platelets used for L-GF^Equina^ production is subjected to pathogen inactivation by UV radiation and riboflavin (vitamin B2). After testing, the final product was proven to be free from aerobic and anaerobic bacteria and fungi. The activation of platelets in vitro prior to lyophilization led to a release of supra-physiological doses of growth factors. Among these were transforming growth factor beta, platelet derived growth factor, insulin like growth factor, and fibroblastic growth factor. Each vial contained platelet derived growth factors equivalent to those found in platelets from 20 mL of whole blood, and platelet counts 2–3 times that of the normal base line.

### 2.4. Assignment of Groups and Protocol of Administration

The mares were selected according to the reproductive history of infertility and if they had undergone repeat breeding for three successive cycles after natural mating. Before the start of the experiment, the mares were gynecologically examined via transrectal palpation and transrectal ultrasonography (SonoScape A5V, SonoScape Medical Crop, Shenzhen, China) to determine the state of the uterus and ovarian follicles, and to find any pathological affection. Uterine swabs were taken from mares under aseptic conditions for bacteriological examination, and free cases were confirmed. Two days after the end of the estrous phase and before treatments, the perineal region of each mare was cleaned and rinsed with clean water and antiseptics and was then dried with tissue paper. The mares were randomly allocated into three groups, as follows: the first group served as a non-treated control group without any interference (*n* = 32), while the second group was treated 20 mL fresh PRP (PRP group, *n* = 32). The third group (L-GF^equina^, *n* = 9) was treated with 20 mL of lyophilized L-GF^equina^ (Cairo Medical Centre Blood Bank, Cairo, Egypt) reconstituted in 20 mL normal saline solution (0.9% NaCl), infused into the uterine body via a one-way uterine catheter [16]. The limited number of mares in the third group was as a result of the limited amount of lyophilized L-GF^equina^ prepared. The intrauterine infusion of PRP or L-GF^equina^ was done using sterilized equipment 2 days after the end of the estrus phase.

### 2.5. Mating and Pregnancy Diagnosis

After treatment, the mares were teased daily for estrus, and the follicular dynamics were monitored during the estrus phase in all the groups via trans-rectal ultrasound. On the fifth day of post treatment, the estrus phase was determined by owner history (winking), the follicular diameter, and the endometrial thickness or edema (column in millimeters in the region of the bifurcation of the uterine horns). If the follicular diameter was larger than 40 mm, together with endometrial grade 2 edema (min. grade 0 and max. grade 4), the induction of ovulation was performed with the intravenous injection of 2500 IU hCG (Pregnyl, Misr Company, Cairo, Egypt) 12–24 h prior to service. Natural insemination was allowed via registered purebred Arabian stallions with a known fertility, 12–24 h after the induction of ovulation.

**Figure 1:**
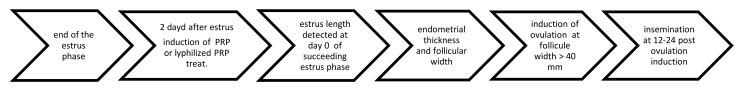
Diagram showing experimental procedures for the conducted work.

Animals treated with care after mating and ultrasonography were examined after insemination after 24 h to confirm successful ovulation. A pregnancy diagnosis was conducted via trans-rectal ultrasound 30 days post insemination to determine the presence of an embryonic vesicle (Figure 1).

### 2.6. Statistical Analysis

The data were enrolled in statistical analysis using SAS (Statistical Analysis System, Version 9.3 for Windows; SAS Institute, Cary, NC, USA). One-way ANOVA procedures followed with LSD as the mean separation test were used to detect the effect of intrauterine infusion of equine PRP or L-GF^equina^ on the estrus cycle length, follicular diameters, and endometrial thickness of the experimental mares. The generalized linear model estimating equation (GEE) in a logistic regression procedure was used to investigate the effect of intrauterine infusion of the PRP or L-GF^equina^ on the pregnancy of purebred Arabian mares. Model specifications included a binomial distribution and logit link function.

## 3. Results

The effect of the intrauterine infusion of PRP or L-GF^equina^ on the estrous cycle length, dominant follicle diameter, and endometrial thickness is presented in Table 1. The results showed that an intrauterine infusion of 20 mL of L-GF^equina^ in repeat breeding Arabian mares produced a significantly (*p* < 0.00) shorter time to the estrus phase (7.56 days) compared with the PRP (9.62 days) or control groups (21.06 days). In addition, the estrous cycle was significantly (*p* < 0.00) shorter in the PRP group compared with the control group. No significant differences were observed for the dominant follicle diameter on the fifth day of the estrus phase between treatments. Neither age or parity showed a significant difference between different experimental groups.

**Figure 2:**
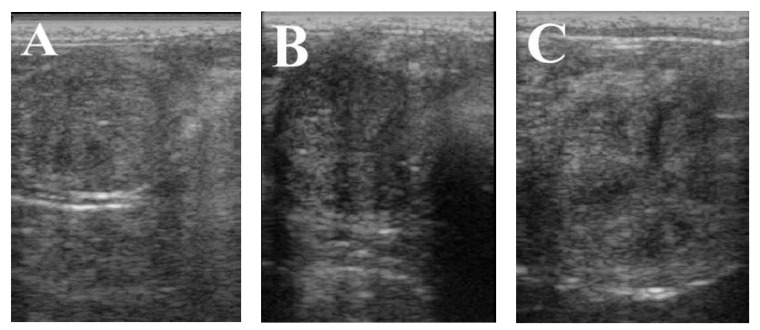
Ultrasonography image for uterine horn thickness on day 5 of the estrus phase in the control (**A**), platelets-rich-plasma (PRP) (**B**), and L-GF^equina^ (**C**) treated groups of pure Arabian mares who experienced repeat breeding.

Concerning the effect of L-GF^equina^ or PRP on endometrial thickness, data the indicate that endometrial thickness was significantly (*p* < 0.01) higher in the PRP treated group (7.33 mm) compared with the L-GF^equina^ (6.86 mm) or control group (5.87). In addition, endometrial thickness was significantly (*p* < 0.01) higher in the L-GF^equina^ group compared with control group (Figure 2).

Table 2 illustrates the effect of an intrauterine injection of L-GF or PRP on the pregnancy rate in repeat breeder Arabian mares. Data show that L-GF^equina^ administration significant increased (*p* < 0.01) the pregnancy rate (66.7%) in repeat breeder Arabian mares compared with the PRP treated group (50%). Moreover, out of 32 PRP treated mares, 16 mares became pregnant, with a pregnancy rate of 50%, which was also significantly (*p* < 0.01) higher than that in the control group (4 out 32 mares, 6.25%).

## 4. Discussion

Currently, the use of platelet-rich plasma (PRP) is progressively increasing in veterinary medicine, especially in horses, as it is used in orthopedic surgery; for the repair of muscles, tendons, and ligaments; for the reversal of skin ulcers [21]; and in chronic degenerative endometritis cases [15]. In addition, researchers have suggested that immunological factors are an important cause of idiopathic infertility [22]. The intrauterine infusion of PRP has many advantages, such as a lower cost, safer use, and no side effects in comparison with other immunomodulatory therapies [15,16]. Therefore, the current investigation aimed to identify the effect of PRP or L-GF^equina^ on the reproductive potential in repeat breeding Arabian mares. Surprisingly, the intrauterine infusion of PRP or L-GF^equina^ two days after the end of estrus phase significantly decreased the duration of the estrous cycle to 7.56 days and 9.63 days for the L-GF^equina^ and PRP groups, respectively, compared with the control group. Short estrous cycles (7–10 days) have previously been reported in cattle and sheep during puberty, first spontaneous and gonadotrophin-induced ovulations postpartum, and at the start of the breeding season in anestrous ewes [23]. It is always associated with decreased concentrations of progesterone after days 5–6 compared with animals with a normal corpus luteum (CL) lifespan [24] and occur as a result of premature endometrial PGF2α release [25]. After exposure to estradiol-17ß (E2), decreasing progesterone allows the oxytocin receptor (OR) to be synthesized, and binds oxytocin to its receptor, releasing PGF2α, and this initiates luteal regression [26]. To the best of our knowledge, no information is available about short luteal phases in horses. Therefore, the explanation of this result is that the high growth factor content of both PRP and L-GF^equina^ could initiate new follicular development and growth, which secretes high E2 that downregulates the progesterone receptor (PR), activating OR and the release of premature PGF2α from the endometrium, leading to premature regression of the CL. These results need further studies to explore the mechanisms of short luteal function in PRP-treated mares on a large scale. However, this study was conducted on a limited number of mares treated with lyophilized L-GF^equina^ prepared.

Furthermore, the obtained results revealed no significant differences between PRP, L-GF^equina^ treated mares, and control mares regarding the diameter of mature Graafian follicles. Similarly, platelet lysate induced a higher follicular survival with no effect follicle development during the in-vitro culture of isolated mouse secondary follicles [27]. In contrast, PRP improved ovarian function in cows with ovary hypofunction; this effect may be as a result of a reduction in follicular atresia or from revitalization of the dormant oocytes [28]. The positive effect of PRP or L-GF^equina^ on follicular growth might be related to the presence of various growth factors that support the early stage of follicle development [29]. On the contrary, PRP has a dominant positive effect on the ovarian cortex volume, pre-antral follicle number, and antral follicle diameter, and decreased the oocyte diameter in pre-antral follicles in infertile rats [30]. The difference could be because of different supplement sources and concentrations of growth factors in PRP or L-GF^equina^.

Moreover, in the present study, the endometrial thickness was higher in L-GF^equina^ and PRP repeat breeder mares than in the control group. In addition, the pregnancy rate was higher in L-GF^equina^ (66.7%) and in PRP treated mares (50%) than in the control group (6.25%). The higher conception rate in the L-GF^equina^ group than in the PRP group could be due to the higher concentration of growth factors than in PRP group. In accordance, Segabinazzi et al. (2017) [16] revealed that the intrauterine PRP infusion decreased the inflammatory response and increase the fertility and conception rate in mares suffering from persistent mating-induced endometritis. Reghini et al. (2016) [15] suggested modulation of the immune response and the prevention of intrauterine fluid retention in mares sufferring from chronic degenerative endometritis via intrauterine infusion of PRP. In addition, Metcalf (2014) [31] reported a significantly high pregnancy rate and that the accumulation of intrauterine fluid was significantly decreased in the PRP treated mares compared with the control ones. These outcomes are also in accordance with previous reports in women [32,33]. In women with recurrent implantation failure, intrauterine autologous PRP infusion increased endometrial thickness, implantation, clinical pregnancy, and live birth rates [34,35]. These results could be due to the downregulation of the intrauterine pro-inflammatory cytokines (IL-1b, IL-6, and IL-8) after insemination in repeat breeding endometritis susceptible mares treated with PRP [36]. In bovines, in vivo treatment with PRP increased the concentration of progesterone receptors and increased uterine cell proliferation and induced a significant increase in the expression of genes involved in the regulation of the estrous cycle and fetal maternal interaction [37]. The bioactivity of PRP or L-GF^equina^ of the genomic, transcriptomic, proteomic, metabolomic, cytokines, growth factors, hormones, and the embryo itself are involved in the mechanism of endometrial receptivity [38]. On the other hand, in a pilot study, PRP had no significant effect on endometrial thickness [39]. This discrepancy may be due to the concentration of platelets or growth factors present in PRP or L-GF^equina^ preparation.

## 5. Conclusions

It is obvious that the infertility of mares and the failure to respond to previous antibiotic therapy is a big problem and is not economically viable when breeding purebred Arabian mares. However, the intrauterine infusion of equine platelet-rich plasma can be used in repeat breeder mares, as it shortens the estrous cycle with no significant effect on the follicular diameter, increasing the endometrial thickness and pregnancy rate in Arabian purebred mares suffering from infertility. Additional studies are ongoing to determine the effects of PRP on fertility rates in these mares.

## Figures and Tables

**Table 1:** Effect of platelet-rich plasma (PRP) treatment on the mature Graafian follicular diameter, endometrial thickness, and estrus cycle length of Arabian mares.

Parameters	Control	PRP	L-GF^equina^	SEM	*p*-Value	Sig.
Age	10.13	8.69	10.33	0.41	0.20	No
Parity	3.99	2.80	3.64	0.22	0.82	No
Graafian follicular diameter (mm)	41.68	42.21	43.30	0.04	0.06	No
Endometrium thickness (mm)	5.87 ^c^	7.33 ^a^	6.86 ^b^	0.31	0.00	Yes
Estrous cycle length (day)	21.06 ^a^	9.63 ^b^	7.56 ^c^	0.05	0.00	Yes

^a,b,c^ Superscript within the same row differ significantly at *p* < 0.05.

**Table 2:** Effect of the PRP treatment on the pregnancy rate of Arabian mares.

Group	N	Pregnant Mares	Pregnancy Rate (%)	Odds Ratio	95% CI ^1^	*p*-Value
Control	32	4	6.25	1	1	0.01
PRP	32	16	50	7.00	1.99–24.58	
L-GF^equina^	9	6	66.67	14.00	2.46–79.55	

^1^ CI = confidence interval.

## Data Availability

The data presented in this study are available on request from the corresponding author.
